# Simultaneous integrated boost (SIB) radiation therapy of right sided breast cancer with and without flattening filter - A treatment planning study

**DOI:** 10.1186/s13014-016-0687-6

**Published:** 2016-08-31

**Authors:** Johannes Maier, Bernadette Knott, Manuel Maerz, Rainer Loeschel, Oliver Koelbl, Barbara Dobler

**Affiliations:** 1 Department of Radiotherapy, Regensburg University Medical Center, Regensburg, Germany; 2 Ostbayerische Technische Hochschule Regensburg, Faculty of Computer Science and Mathematics, Regensburg, Germany

## Abstract

**Background:**

The aim of the study was to compare the two irradiation modes with (FF) and without flattening filter (FFF) for three different treatment techniques for simultaneous integrated boost radiation therapy of patients with right sided breast cancer.

**Methods:**

An Elekta Synergy linac with Agility collimating device is used to simulate the treatment of 10 patients. Six plans were generated in Monaco 5.0 for each patient treating the whole breast and a simultaneous integrated boost (SIB) volume: intensity modulated radiation therapy (IMRT), volumetric modulated arc therapy (VMAT) and a tangential arc VMAT (tVMAT), each with and without flattening filter. Plan quality was assessed considering target coverage, sparing of the contralateral breast, the lungs, the heart and the normal tissue. All plans were verified by a 2D-ionisation-chamber-array and delivery times were measured and compared. The Wilcoxon test was used for statistical analysis with a significance level of 0.05.

**Results:**

Significantly best target coverage and homogeneity was achieved using VMAT FFF with V_95%_ = (98.7 ± 0.8) % and HI = (8.2 ± 0.9) % for the SIB and V_95%_ = (98.3 ± 0.7) % for the PTV, whereas tVMAT showed significantly lowest doses to the contralateral organs at risk with a D_mean_ of (0.7 ± 0.1) Gy for the contralateral lung, (1.0 ± 0.2) Gy for the contralateral breast and (1.4 ± 0.2) Gy for the heart. All plans passed the gamma evaluation with a mean passing rate of (99.2 ± 0.8) %. Delivery times were significantly reduced for VMAT and tVMAT but increased for IMRT, when FFF was used. Lowest delivery times were observed for tVMAT FFF with (1:20 ± 0:07) min.

**Conclusion:**

Balancing target coverage, OAR sparing and delivery time, VMAT FFF and tVMAT FFF are considered the preferable of the investigated treatment options in simultaneous integrated boost irradiation of right sided breast cancer for the combination of an Elekta Synergy linac with Agility and the treatment planning system Monaco 5.0.

## Background

Adjuvant radiation therapy following breast conserving surgery allows improving local control and overall survival in early stage breast cancer patients [1]. Treatment techniques for radiation therapy have been established ranging from conventional tangential fields over intensity modulated radiation therapy (IMRT) to volumetric modulated arc therapy (VMAT), and been compared in various planning studies [2–10]. Cardiac sparing in patients with left sided breast cancer has been considered the main advantage of IMRT and VMAT as compared to tangential fields [2–7, 11]. A few planning studies include right sided as well as left sided breast cancer [8–10], but dedicated planning studies for right sided breast cancer seemed not to be of interest up to now. During the last years, however, simultaneous integrated boost radiation therapy superseded sequential boost therapy as the standard fractionation scheme [12, 13]. In this case, IMRT and VMAT have shown advantages in target coverage and sparing of the organs at risk (OAR) as compared to 3D-CRT techniques [10, 14], thus becoming more important also for right sided breast cancer.

The latest development in the technology of linear accelerators is the opportunity to irradiate patients without a flattening filter in the beam path to increase dose rate and reduce beam-on times as well as out-of-field doses [15]. Two planning studies have already been published comparing the two irradiation modes for breast cancer, both of them dealing with left sided breast cancer [5, 7]. Since dose volume restrictions to the heart might, however, be limiting the reduction of dose to the other organs at risk to a larger extent in the therapy of left sided breast cancer, results cannot simply be transferred to right sided breast cancer cases. Dedicated planning studies for radiation therapy of right sided breast cancer are therefore necessary to identify the optimal treatment technique also for this group of patients.

The aim of the study presented here was to compare the two irradiation modes with (FF) and without flattening filter (FFF) for three different treatment techniques for simultaneous integrated boost radiation therapy of patients with right sided breast cancer.

## Methods

### Patients

CT data of 10 patients with right sided breast cancer were randomly selected from our database for a retrospective planning study. The target volumes were delineated by a clinical oncologist according to Dellas et al. [16]. The PTV includes the whole right mammary gland without parasternal, axial or supraclavicular lymph nodes. All patients received a simultaneous boost irradiation to the tumour bed. The volume for the boost therapy was defined with the help of preoperative information (results of mammography, ultrasound and clinical examination) and the surgical report. Moreover the scar, the presence of surgical clips in the tumour bed, and changes within the breast tissue following surgery were important information for the definition of the boost volume. To create the simultaneous integrated boost planning target volume (SIB) a safety margin was added to the boost volume. This safety margin was constrained to be inside the patient outline.

Target volumes ranged from 454 ccm to 1377 ccm for the PTV, and from 32 ccm to 172 ccm for the SIB. The treatment goals were an average dose of 50.4 Gy in 28 fractions of 1.8 Gy in the PTV and 63 Gy in 28 fractions of 2.25 Gy in the SIB according to Sedlmayer et al. [17]. For the organs at risk (OAR) the following dose volume tolerances were chosen as clinical goals: The volume of the ipsilateral lung V_20Gy_ receiving ≥ 20 Gy should be < 15 %, the mean dose < 12 Gy [18]. The volume of the contralateral lung V_5Gy_ should be restricted to 5 % [19], the mean dose to 3 Gy [10]. The mean dose to the heart should be kept below 5 Gy [10, 18]. For the contralateral breast the mean dose should be < 3 Gy [10].

### Linear accelerator and treatment planning system

Treatment planning was performed with Monaco 5.0 (Elekta AB, Stockholm, Sweden) with X-ray Voxel Monte Carlo (XVMC) dose algorithm for a Synergy linear accelerator with Agility collimating device (Elekta AB, Stockholm, Sweden) and 6 MV photons with flattening filter or without, in the following referred to as FF and FFF beams. The FFF beams were energy-matched to the FF beams as it is common for Elekta accelerators [20–23]. This means, that the parameters of the accelerator were tuned for the FFF mode to achieve the same percentage depth dose at 10 cm depth for a 10 cm × 10 cm field and the same quality index as in FF mode. The agreement of both parameters was within 1 %. The linac was calibrated such that 100 MU correspond to 1 Gy at the central axis at depth dose maximum for a 10 cm × 10 cm field with a source surface distance of 100 cm for both irradiation modes. The multi leaf collimator (MLC) consists of 80 leaf pairs of 5 mm width at isocenter. The transmission was 0.6 % for both irradiation modes. The maximum nominal dose rate is 500 MU/min in FF mode and 1700 MU/min in FFF mode. Due to limitations in the speed of mechanical movements, the maximum dose rate is, however, not applied throughout VMAT treatments. Beam profiles, depth doses and dose output were found to be stable for 4 MU and larger in both irradiation modes as also reported by Akino [24]. Verification of the linac model in Monaco 5.0 with XVMC dose calculations of absolute dose, percentage depth doses, profiles, and output factors was within specifications for both FF and FFF. The specifications were 2 % of calibration dose in low dose gradient areas (high and low dose) and 3 mm distance to agreement in sharp dose gradients.

### Treatment planning

In total six treatment plans were created for each patient, using the three different treatment techniques IMRT, VMAT and tangential arc VMAT (tVMAT) each with and without flattening filter. The IMRT plans were realized as step and shoot IMRT and consisted of 10 beams with gantry angles of 55°, 35°, 15°, 355°, 335°, 315°, 285°, 250°, 225°, and 200°. The VMAT plans were realized with two arcs covering gantry angles from 180° to 60° clockwise (cw) and counter-clockwise (ccw). For the tVMAT two partial arcs were used ranging from 60° to 5° and from 255° to 200° gantry angle ccw similar to the technique described by Pasler et al. [4]. All plans were optimized in Monaco 5.0 using identical constraints. The optimization in Monaco is based on biological objective functions rather than on physical dose volume objectives. Therefore the constraints used for optimization are not identical to the physical dose volume tolerances derived from literature and listed in the section “Patients” and in Table 1 in the column “Goal”. In Monaco, the constraints to the OAR are prioritized over the constraints to the targets. Therefore suitable constraints for optimization were determined creating plans in FF mode lowering OAR doses as much as possible without compromising target coverage and homogeneity. The identical constraints were then also used for optimization of the FFF plans. A so called “surface margin” of 3 mm was used to prevent the optimizer from adding dose to the surface region to compensate for the buildup effect. To ensure coverage of the breast a so called “auto flash margin” of 2 cm was used, which automatically extends the beam 2 cm beyond the patient outline. For IMRT the minimal number of monitor units (MU) per segment was set to 4 due to the determined stability of the beam for 4 MU and higher. The minimal segment area was limited to 4 cm^2^. For VMAT the number of control points per arc was limited to 120, for IMRT a straight limitation of control points was not possible in this version of Monaco. The dose calculation was performed with a grid size of 3 mm and the statistical uncertainty parameter was set to 1 %. All plans were normalized to the mean dose in the SIB. In the following the plans are referred to as IMRT FF, IMRT FFF, VMAT FF, VMAT FFF, tVMAT FF, and tVMAT FFF.

**Table 1:** Comparison of plan quality

Parameter		Goal	IMRT FF	IMRT FFF	VMAT FF	VMAT FFF	tVMAT FF	tVMAT FFF
SIBm5	V_95%_	>95	97.2 ± 1.2	97.5 ± 1.3	98.2 ± 1.0	***98.7 ± 0.8***	95.5 ± 2.2	95.7 ± 2.0
	D_98%_		59.6 ± 0.4	59.7 ± 0.4	60.0 ± 0.5	***60.2 ± 0.4***	59.0 ± 0.7	59.1 ± 0.6
	D_02%_		65.6 ± 0.4	65.6 ± 0.2	65.5 ± 0.4	65.3 ± 0.4	66.1 ± 0.4	66.1 ± 0.5
	HI		9.6 ± 1.2	9.3 ± 0.9	8.8 ± 1.2	***8.2 ± 0.9***	11.3 ± 1.4	11.1 ± 1.5
	CI	>0.7	0.77 ± 0.05	0.78 ± 0.05	0.78 ± 0.05	0.78 ± 0.05	0.75 ± 0.05	0.75 ± 0.05
PTVm5	V_95%_	>95	96.8 ± 2.3	96.8 ± 1.9	97.6 ± 1.5	***98.3 ± 0.7***	95.2 ± 4.3	94.9 ± 3.8
	CI	>0.7	0.77 ± 0.05	0.77 ± 0.06	0.77 ± 0.05	0.78 ± 0.05	0.75 ± 0.04	**0.76 ± 0.04**
PTV (excluding SIB)	D_98%_		46.9 ± 0.7	46.9 ± 0.6	46.9 ± 1.1	***47.4 ± 0.6***	45.7 ± 2.0	45.5 ± 2.0
	D_02%_		55.2 ± 0.5	55.1 ± 0.3	55.4 ± 0.4	55.4 ± 0.4	56.1 ± 0.7	56.1 ± 0.7
	HI		16.4 ± 1.6	16.3 ± 1.1	16.9 ± 2.2	15.8 ± 1.1	20.4 ± 4.5	20.9 ± 4.6
Lung ipsilateral	V_20Gy_	<15	13.2 ± 3.8	13.1 ± 3.6	13.1 ± 3.3	13.5 ± 3.3	13.8 ± 3.1	14.1 ± 3.0
	D_mean_	<12	8.7 ± 1.3	8.7 ± 1.3	8.7 ± 1.2	8.9 ± 1.2	8.9 ± 1.0	8.9 ± 1.0
Lung contralateral	V_5Gy_	<5	**0.2 ± 0.4**	0.4 ± 0.4	0.1 ± 0.2	0.3 ± 0.3	0.0 ± 0.0	0.0 ± 0.0
	D_mean_	<3	**1.6 ± 0.4**	1.7 ± 0.2	1.7 ± 0.2	1.8 ± 0.2	1.0 ± 0.1	***0.7 ± 0.1***
Breast contralateral	D_mean_	<3	1.8 ± 0.4	1.9 ± 0.4	1.8 ± 0.3	1.8 ± 0.4	1.3 ± 0.2	***1.0 ± 0.2***
	D_02%_		**5.4 ± 1.3**	5.6 ± 1.2	4.5 ± 1.0	4.8 ± 1.2	3.2 ± 0.6	***2.8 ± 0.8***
Heart	D_mean_	<5	2.4 ± 0.5	2.6 ± 0.4	2.7 ± 0.5	2.6 ± 0.5	1.7 ± 0.3	***1.4 ± 0.2***
	D_02%_		5.0 ± 1.1	5.7 ± 0.9	5.0 ± 1.0	5.1 ± 0.9	3.3 ± 0.5	***3.0 ± 0.6***
Normal Tissue	D_mean_		4.4 ± 0.4	4.6 ± 0.3	4.4 ± 0.4	4.4 ± 0.3	3.8 ± 0.3	***3.6 ± 0.3***
	D_02%_		42.5 ± 1.1	**42.2 ± 1.3**	41.9 ± 1.7	41.9 ± 1.3	41.9 ± 2.2	41.5 ± 1.9

Mean values and standard deviation of the dose volume parameters for FF and FFF mode averaged over all patients separated by the treatment technique. Dose values are given in Gy, volumes in % of the structure volume. HI stands for homogeneity index (values are reported in %), CI for conformity index. Bold values indicate statistically significant superior values in the comparison of FF vs FFF. Bold-italic letters indicate best values in the comparison of all planning techniques and irradiation modes

### Dosimetry

**Fig. 1:**
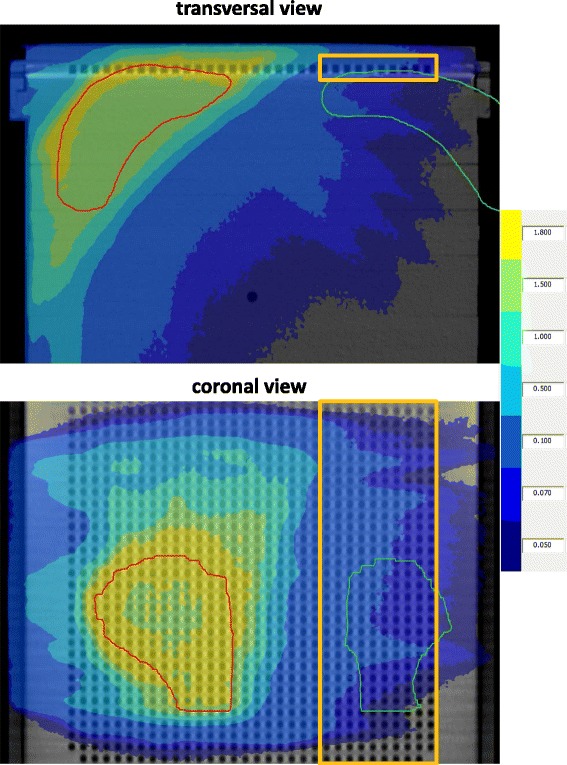
Measurement setup for verification of the low dose. Transversal (*top*) and coronal (*bottom*) CT slice of the phantom used for verification of the dose in the low dose region. The isocenter was placed such that the high dose area of the PTV (*red*) was inside the phantom to maintain phantom scatter and the dose region corresponding to the medial part of the contralateral breast (*green*) was located in the active area of the MatriXX Evolution. Isodoses are given in Gy. The *orange* box shows the ROI used for evaluation of the low dose region

For verification all 60 plans were transferred to a CT scan of the MatriXX Evolution™ 2D-ionisation-chamber-array (IBA Dosimetry, Schwarzenbruck, Germany) set up in between one 10 cm (bottom) and one 9.7 cm (top) stack of RW3 slabs (PTW, Freiburg, Germany) for measurement in a coronal plane [25, 26]. Measurements were corrected for angular dependencies and couch attenuation in the software OmniPro I’mRT v.1.7a (IBA Dosimetry, Schwarzenbruck, Germany) which is steering the measurement and used for evaluation. Gamma indices as defined by Low et al. [27] were calculated with a dose tolerance of 3 % of the maximum dose and 3 mm distance to agreement. Dose calculations are considered acceptable if at least 95 % of the pixels with a dose value of ≥ 10 % of the maximum dose have a gamma value ≤ 1 as recommended by the AAPM TG119 [28, 29]. For verification of the dose calculation in the low dose region a second setup was used with only 1 cm RW3 buildup on top of the MatriXX Evolution™. The isocenter was placed such that the high dose area of the PTV was inside the phantom to maintain phantom scatter and the dose region corresponding to the medial part of the contralateral breast was located in the active area of the MatriXX Evolution™. A region of interest (ROI) was defined in the software OmniPro I’mRT in the low dose area corresponding to the contralateral breast for evaluation as illustrated in Fig. 1 by the orange rectangle. The size of the rectangle was about 8 cm in width and the full size of the active area of the MatriXX in cranio-caudal direction. Dose differences between measured and calculated doses in the ROI were calculated and compared for the different techniques.

### Efficiency

Delivery times were measured from first beam on to last beam off to assess the achievable reduction in total delivery time. In addition the number of required monitor units (MU) and control points was compared.

### Evaluation

Plan quality was assessed by analysis of the dose volume histogram (DVH) with respect to target coverage, dose homogeneity and conformity, dose to the organs at risk and normal tissue as described in the treatment goals. For evaluation the PTV and SIB were cropped by 5 mm from the external contour (PTVm5 and SIBm5) as it is common to account for the buildup effect [5, 7]. Target coverage was represented by the volume of PTVm5 and SIBm5 covered by 95 % of the prescription dose (V_95%_). The homogeneity index was defined as HI := (D_2%_ - D_98%_)/D_50%_ according to ICRU 83 [30] and reported in percent, the conformity index according to Paddick et al. [31] as CI := V_95%_^2^ / (TV ⋅ PIV). Here TV means the volume of the respective target, PIV the total volume covered by 95 % of the prescription dose in the respective target. Minimum and maximum doses were represented by the dose to 98 and 2 % of the volume (D_98%_ and D_02%_) according to ICRU recommendations [30]. The homogeneity index, D_98%_ and D_02%_ were recorded for SIBm5 and for PTVm5 excluding the SIB extended by a 5 mm margin, to exclude the high dose region of the SIB from the analysis of the PTV. The DVH parameters recorded for the OAR were D_mean_ for all OAR and V_20Gy_ for the ipsilateral lung, V_5Gy_ for the contralateral lung, and D_02%_ for the contralateral breast, the heart and the normal tissue.

The Wilcoxon test implemented in IBM SPSS® Statistics 23.0 (IBM Corporation) was used for statistical analysis with a significance level of 0.05. Statistically significant differences are reported in the text.

## Results

### Plan quality

**Fig. 2:**
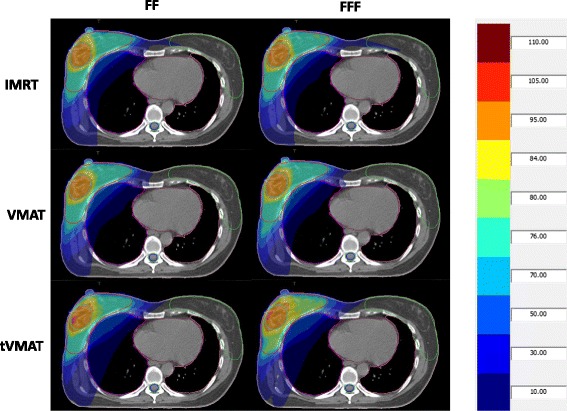
Comparison of dose distributions of FF versus FFF for all techniques. Comparison of dose distributions for all treatment techniques and irradiation modes in one transversal slice for a sample case. The PTV is delineated in *red*, SIB bright *blue*, ipsilateral lung *magenta*, contralateral lung *pink*, heart *brown*, contralateral breast *green*, spinal cord *blue* and spinal canal *green*. Isodoses are given in percent of the prescription dose to the SIB, i.e. 63 Gy. The *orange* band shows 95 % of the prescription dose to the SIB, the bright *blue* the 95 % of the prescription dose to the PTV

**Fig. 3:**
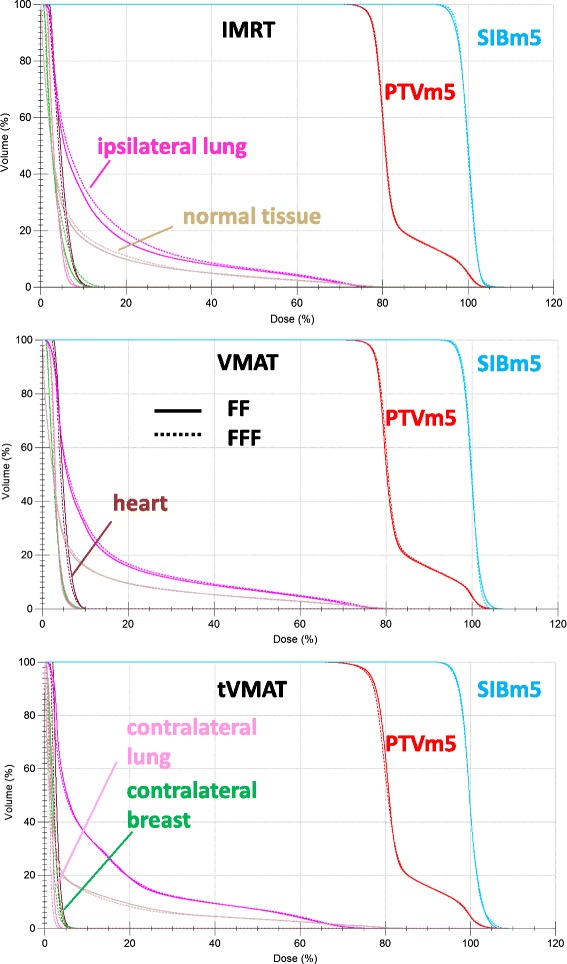
Comparison of dose volume histograms of FF versus FFF for all techniques. Comparison of dose volume histograms for the case of Fig. 2. Solid lines show DVH of the FF plan, dotted lines of the corresponding FFF plan. PTVm5 is drawn in *red*, SIBm5 bright *blue*, ipsilateral lung *magenta*, contralateral lung *pink*, heart *brown*, contralateral breast *green*, normal tissue *beige*. The spinal cord and spinal canal are not shown for the sake of clarity

Details about DVH parameters averaged over all patients are given in Table 1. A comparison of dose distributions and corresponding dose volume histograms for FF versus FFF mode for all treatment techniques is shown in Figs. 2 and 3 for a case with medium sized targets. The results listed in Table 1 show that FFF led to significantly superior results in plan quality when VMAT or tVMAT were used as treatment technique: VMAT FFF improved target coverage and homogeneity significantly keeping OAR doses comparable to VMAT FF. tVMAT FFF reduced OAR doses significantly without compromising target coverage and homogeneity as compared to tVMAT FF. For IMRT, on the contrary, FF showed better results, since only one significant difference was in favour of FFF as compared to three in favour of FF. Differences between the irradiation modes were, however, in general small even though statistically significant, and OAR doses were mostly below the goal in both treatment modes.

**Fig. 4:**
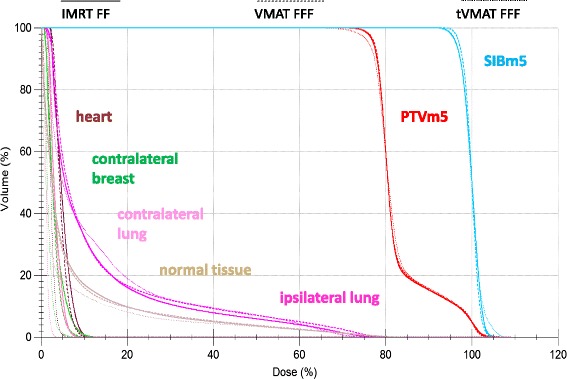
Comparison of dose volume histograms of different treatment techniques. Comparison of dose volume histograms of the different treatment techniques for the case of Fig. 2. For the sake of clarity only the superior plan is shown for each technique, i.e. IMRT FF, VMAT FFF and tVMAT FFF. PTVm5 is drawn in *red*, SIBm5 bright *blue*, ipsilateral lung *magenta*, contralateral lung *pink*, heart *brown*, contralateral breast *green*, normal tissue *beige*

For comparison of the planning techniques, the statistically superior plan of each technique was chosen, i.e. IMRT FF, VMAT FFF and tVMAT FFF. VMAT FFF showed over all best target coverage and homogeneity with similar doses to the OAR and the normal tissue as compared to IMRT FF. tVMAT FFF showed lowest doses to the contralateral OAR at the cost of reduced target coverage and homogeneity as compared to both VMAT FFF and IMRT FF. The goal of V_95%_ > 95 % in the SIBm5 was achieved in only 50 % of the cases for tVMAT FFF as compared to 100 % of the cases for VMAT FFF. Figure 4 shows a comparison of the dose volume histograms of the superior plan of each treatment techniques for the patient of Figs. 2 and 3.

### Dosimetry

All 60 plans passed the gamma evaluation in the measurement setup 1 with a mean passing rate of (99.2 ± 0.7) % for FF and (99.1 ± 0.8) % for FFF plans. Passing rates were > 97.0 % in all cases i.e. well above the tolerance of 95 %. Average doses in the low dose ROI in setup 2 were (20.1 ± 7.8) cGy (measured) and (19.7 ± 7.7) cGy (calculated). The verification of the dose calculation in this low dose region showed large differences between the treatment techniques: High agreement between calculations and measurements could be observed for VMAT and tVMAT plans both in FF and FFF mode, with dose deviations of (0.3 ± 2.0) %, (0.2 ± 1.6) %, (0.1 ± 2.1) % and (0.6 ± 1.8) % of the average dose in the ROI for VMAT FF, VMAT FFF, tVMAT FF and tVMAT FFF respectively. This corresponds to dose deviations of well below ± 1.0 cGy. For IMRT plans, however, dose deviations were significantly higher with (−6.0 ± 2.0) % in FF mode and (−9.3 ± 3.0) % in FFF mode, but still in the range of 1 % of the prescription dose. Negative signs mean an underestimation of dose in the dose calculation.

### Efficiency

Treatment times were significantly reduced by 7 % for VMAT and 32 % for tVMAT in FFF mode as compared to FF. IMRT FFF, on the contrary, showed on average 8 % longer delivery times than IMRT FF, differences were, however, not statistically significant in this case. Comparison over all treatment techniques showed lowest treatment times for tVMAT FFF, with a reduction by 86 % as compared to IMRT FF and by 46 % as compared to VMAT FFF. Details are listed in Table 2.

**Table 2:** Comparison of plan delivery

Plan	Time [min:sec]	MU	Control points
IMRT FF	09:29 ± 1:25	630 ± 114^a^	188 ± 31^a^
IMRT FFF	10:13 ± 1:30	792 ± 128	228 ± 39
VMAT FF	2:38 ± 0:17	713 ± 77^a^	162 ± 10^a^
VMAT FFF	**2:27 ± 0:14**	852 ± 105	181 ± 7
tVMAT FF	1:58 ± 0:14	651 ± 124^a^	53 ± 2
tVMAT FFF	***1:20 ± 0:07***	763 ± 96	52 ± 2

Mean values and standard deviation of delivery time, monitor units and control points for FF and FFF mode averaged over all patients separated by the treatment technique. Bold values indicate statistically significant superior values in the comparison of FF vs FFF. Bold-italic letters indicate best values in the comparison of all planning techniques and irradiation modes. ^a^indicates statistical significance in the comparison of FF vs FFF without judgement, since the number of monitor units and the number of control points are no measures of quality

## Discussion

The aim of this study was to investigate the potential of the flattening filter free (FFF) mode of a linear accelerator for three different treatment techniques for patients with right sided breast cancer. The results of the study show, that FFF led to significantly superior results with regard to plan quality, when VMAT or tVMAT were used as planning technique, whereas the opposite was the case for IMRT. Differences in plan quality were, however, in general small and the clinical relevance of the differences remains to be shown. The constraints used for optimization were derived from iterative treatment planning in FF mode and used for optimization in FFF mode without adaptation. This approach was chosen for reasons of comparability in order to not bias the results by the use of different constraints. It might be possible to further improve plan quality in FFF mode by adjusting the constraints for the optimization in FFF mode. This is, however, beyond the scope of this study.

Comparison of all treatment techniques and irradiation modes showed significantly best target coverage and homogeneity for VMAT FFF and lowest doses to the contralateral OAR and normal tissue for tVMAT FFF. This has been confirmed by measurements which showed high agreement between measurements and calculations in the low dose region for VMAT and tVMAT as well as in the high dose area corresponding to the target volumes for all plans. No significant differences could be observed in dose calculation accuracy between the two irradiation modes FF and FFF neither in the high nor in the low dose region. Gamma passing rates were high (above 97 %) for each individual plan independently of the treatment technique and irradiation mode. Dose deviations in the low dose region were significantly higher for IMRT than for VMAT and tVMAT in both irradiation modes, but still in the range of 1 % of the prescription dose, which is excellent considering the recommendations of ESTRO for the verification of simple open fields with tolerances of 3 % of the central axis dose or 30 % of the local dose in the low dose region [32]. For verification of complete IMRT plans, a confidence limit of 4 % is recommended [33]. With respect to dose calculation accuracy VMAT and tVMAT are therefore considered the preferred treatment techniques independently on the irradiation mode.

The number of MU and control points was significantly higher in FFF irradiation mode, with an increase by 17 % for tVMAT, 19 % for VMAT and 26 % for IMRT plans. The significantly highest number of MU was observed for VMAT FFF. The increase in MU can be explained by the fact that the linac is calibrated such that 100 MU correspond to 1 Gy under reference conditions on the central beam axis for both FF and FFF. Due to the shape of the dose profile, the dose is lower outside the central axis for FFF beams. Therefore additional MU delivered in smaller off axis segments are required to achieve the same dose away from the central beam in FFF mode. Concerns had been raised that an increase of MU might mitigate the potential advantages of FFF with respect to treatment time and MLC transmission leading to potentially higher OAR and normal tissue doses. The results of our study showed that for VMAT and tVMAT treatment times were significantly reduced by 7 % and 32 % and doses to the OAR were comparable for VMAT and even significantly reduced for tVMAT despite of significantly increased MU in FFF mode. Due to the excellent agreement between dose calculations and measurements for VMAT and tVMAT in the low as well as in the high dose region, the results of the dose volume analysis are considered reliable. For IMRT, longer treatment times and a significant increase in OAR doses were observed in the DVH analysis, normal tissue dose was at the same time significantly reduced. The results of the DVH analysis are, however, somewhat less reliable for IMRT than for VMAT and tVMAT due to the observed uncertainty in dose calculation in the low dose region.

The difference in the influence of irradiation mode on treatment time can be explained by the fact that for step and shoot IMRT delivery times increase with the number of control points due to the interruption of irradiation during movement of the MLC. For VMAT techniques the number of control points does not affect the treatment time, since the beam stays on during MLC movement. The number of MU required in FFF plans increased by a factor of 1.2 whereas dose rates delivered in FFF mode increased by up to a factor of 3.4, leading to shorter treatment times for FFF in VMAT and tVMAT techniques. The maximum dose rate is, however, not applied throughout the whole treatment, due to limitations in the speed of mechanical movements therefore the ratio of treatment time in FFF mode to treatment time in FF mode is in general larger than 1.2: 3.4. In simultaneous integrated boost irradiation using IMRT, VMAT or tVMAT dose conformity is higher as compared to conventional tangential field techniques for whole breast treatment. Therefore reduction of intrafractional movement becomes more important, to avoid shifts of the dose distribution relative to the target due to systematic drifts in patient position [34]. Wiant et al. showed in their study of intrafraction motion of breast cancer patients, “that the patients tend to drift further away from their initial position and they tend to have more short-term random motion as time in the treatment position increases” [35]. The authors observed a linear increase of the mean shift with time during the first 5 min of a fraction. The reduction of the total treatment time observed for tVMAT FFF is therefore corresponding to a reduction of the mean shift by 32 % as compared to tVMAT FF and 46 % as compared to VMAT FFF. With respect to treatment time and reduction of shifts of the dose distributions relative to the target, tVMAT FFF is therefore considered the preferable treatment option. An investigation of plan robustness against intrafractional breathing movement as a function of treatment technique was, however, beyond the scope of the study. It is therefore advised to use any form of motion control in combination with volume imaging to reduce uncertainties due to breathing.

Balancing target coverage, OAR sparing, agreement of dose calculations and measurements, and delivery time, VMAT FFF and tVMAT FFF are considered the preferred treatment options for SIB radiation therapy of right sided breast cancer in this study. Due to the relatively large standard deviations especially in the target coverage for tVMAT plans and in the V_20Gy_ of the ispilateral lung, the clinical decision is depending on the individual patient’s plan.

Two planning studies have been published comparing the two irradiation modes for left sided breast cancer, one of them dealing with whole breast irradiation [5], the other with SIB treatment [7]. Spruijt et al. [7] performed a planning study for SIB treatment of left sided breast cancer comparing FF and FFF for different static field and IMRT techniques planned with Eclipse treatment planning system for a Varian True Beam Linac. They found comparable plan quality and lower delivery times when FFF beams were used. For verification of the dose calculation one phantom case was created for out-of-field dose measurements 0.3 to 3.1 cm from the field edge. They found an average reduction in out-of-field dose of 10 %. Comparison to dose calculations in this region showed, however, an underestimation by the treatment planning system Eclipse of 26 % to 85 % as compared to measurements. Because of the uncertainties in the dose calculation the authors abandoned evaluation of dose volume parameters of the contralateral breast and lung in their planning study. The agreement between calculations and measurements is substantially higher in our study with (0.1 ± 2.1) % to (−9.3 ± 3.0) % dose deviation in the low dose region of around 20 cGy depending on the treatment technique. A comparison of the plan quality achieved in their study to our results is therefore not possible.

Koivumaki et al. [5] compared FF and FFF irradiation for tVMAT and tangential IMRT for whole breast irradiation of left sided breast cancer in a hypofractionated scheme of 15 × 2.67 Gy. Treatment planning was conducted in an earlier version of Monaco v3.0 for an Elekta Infinity linac. The authors found a significant reduction in beam-on time when FFF was used at comparable plan quality for tVMAT and degraded plan quality for the tangential IMRT plans. The IMRT and VMAT techniques used in their study differ from the techniques presented here, but the effect that FFF seems beneficial for VMAT but not for IMRT treatments can be observed in both studies. Comparing the results of both studies in plan quality, the differences in total dose of 40 Gy versus 50.4 Gy and 63 Gy have to be taken into account. Whereas mean doses to the contralateral breast and lung are similar for the tVMAT plans in both studies, the mean and maximum dose to the heart and V_20Gy_ and the D_mean_ of ipsilateral lung are lower in the study presented here. The same trend could be observed in comparison to the results of Pasler et al. [4] who compared tVMAT to VMAT for left sided breast cancer. For the dose to the heart, differences are expected due to the location of the heart. The differences in the ipsilateral lung might be explained by the fact that dose volume restrictions to the heart are limiting the reduction of dose to the ipsilateral lung to a larger extent in the therapy of left sided breast cancer than for right sided breast cancer.

## Conclusions

The use of FFF allowed creating acceptable treatment plan quality for the combination of an Elekta Synergy linac with Agility and the treatment planning system Monaco 5.0 in simultaneous integrated boost irradiation of right sided breast cancer in all three treatment techniques investigated in this study. FFF led to superior results when VMAT or tVMAT was used as treatment technique, whereas for IMRT results were superior in FF mode. Balancing target coverage, OAR sparing, agreement of dose calculations and measurements, and delivery time, VMAT FFF and tVMAT FFF are considered the preferable of the investigated treatment options.

## Abbreviations

CI: Conformity index
D_mean_: Mean dose of a structure
DVH: Dose volume histogram
D_x_: Dose to volume x of the structure
FF: Flattening filter
FFF: Flattening filter free
HI: Homogeneity index
IMRT: Intensity modulated radiation therapy
Min: Minutes
MLC: Multi leaf collimator
PIV: Total volume of the 95 % isodose
PTV: Planning target volume
ROI: Region of interest
TV: Volume of the PTV
tVMAT: Tangential arc VMAT
VMAT: Volumetric modulated arc therapy
V_x%_: Volume covered by x% of the prescription dose
